# A new species of *Chilobrachys* (Araneae, Theraphosidae) from Guangdong, China

**DOI:** 10.3897/BDJ.10.e96467

**Published:** 2022-11-03

**Authors:** Ye-Jie Lin, Shuqiang Li, Chunyan Xie

**Affiliations:** 1 Hebei Key Laboratory of Animal Diversity, College of Life Science, Langfang Normal University, Langfang, China Hebei Key Laboratory of Animal Diversity, College of Life Science, Langfang Normal University Langfang China; 2 Institute of Zoology, Chinese Academy of sciences, Beijing, China Institute of Zoology, Chinese Academy of sciences Beijing China

## Abstract

**Background:**

The theraphosid spider genus *Chilobrachys* Karsch, 1892 contains 30 species, almost entirely limited to Indochina, India, Sri Lanka and China. Six species of *Chilobrachys* are currently known from China: *C.dominus* Lin & Li, 2022 (Yunnan), *C.guangxiensis* (Yin & Tan, 2000) (Guangxi, Hainan), *C.hubei* Song & Zhao, 1988 (Hubei, Chongqing), *C.jinchengi* Lin & Li, 2022 (Tibet), *C.liboensis* Zhu & Zhang, 2008 (Guizhou, Guangxi) and *C.lubricus* Yu et al., 2021 (Yunnan).

**New information:**

A new species, *Chilobrachysqishuoi* Lin & Li, **sp. n.**, is described from Guangdong, China, on the basis of both sexes. This is the easternmost *Chilobrachys* species known. Photos and a morphological description of the new species are provided. Type materials are deposited in the Institute of Zoology, Chinese Academy of Sciences (IZCAS) in Beijing, China.

## Introduction

The spider family Theraphosidae Thorell, 1869, commonly known as tarantulas, currently comprises 1041 species in 156 genera, of which *Chilobrachys* Karsch, 1892 contains 30 species, almost entirely limited to Indochina, India, Sri Lanka and China (Li 2020, Li et al. 2021, Yao et al. 2021, Zhao et al. 2022). Although this genus contains a relatively large number of species, 12 species are described from one sex and few historical species have been restudied. Six valid *Chilobrachys* species are currently known from China: *C.dominus* Lin & Li, 2022 (Yunnan), *C.guangxiensis* (Yin & Tan, 2000) (Guangxi, Hainan), *C.hubei* Song & Zhao, 1988 (Hubei, Chongqing), *C.jinchengi* Lin & Li, 2022 (Tibet), *C.liboensis* Zhu & Zhang, 2008 (Guizhou, Guangxi) and *C.lubricus* Yu et al., 2021 (Yunnan). One previous species from China, *C.tschankhoensis* Schenkel, 1963 was made a *nomen dubium* by Sherwood & Gabriel (2020) (Schenkel 1963, Zhu and Zhang 2008, Sherwood and Gabriel 2020, Yu et al. 2021, Lin et al. 2022, World Spider Catalog 2022).

The most distinctive feature of this genus is the basal, ventrally standing knife-like strikers on the chelicerae and a single or double row of paddle hairs (lyra) overlapped by a fringe of lesser stridulating setae on the maxillae (Raven 1985, Zhu and Zhang 2008). Previous researchers have often overlooked the morphological characteristics of the stridulatory setae of species within the same genus. The terminal structure of embolus also has an important role in species identification.

Here, we describe a new species: *Chilobrachysqishuoi* sp. n. from Qingyuan, Guangdong. It is the easternmost *Chilobrachys* species known.

## Materials and methods

All specimens were preserved in 75% ethanol and the type specimens of *Chilobrachysqishuoi* sp. n. were deposited in the Institute of Zoology, Chinese Academy of Sciences in Beijing (**IZCAS**). Spermathecae were cleared in trypsin enzyme solution to dissolve non-chitinous tissues. Specimens were examined using a LEICA M205C stereomicroscope. Photomicroscopy images were taken with an Olympus C7070 zoom digital camera (7.1 megapixels). Laboratory habitus photographs were taken with a Sony A7RIV digital camera equipped with a Sony FE 90 mm Goss lens. Photos were stacked with Helicon Focus (v. 7.6.1) or Zerene Stacker (v. 1.04) and processed in Adobe Photoshop CC2022.

All measurements are in millimetres and were obtained with an Olympus SZX16 stereomicroscope with a Zongyuan CCD industrial camera. Body length was measured without chelicerae. Eye sizes were measured as the maximum diameter from either the dorsal or frontal view. The largest stridulatory seta was selected as the sample. The length of the contraction area is defined as the length from the position on the stalks that is as wide as the spermathecal lobe to the neck. The length of the spermathecal lobe is defined as the length from the neck to the terminal of the spermathecal lobe. The length of stalks is defined as the length from the neck to the end of the receptacles. Leg measurements are given as follows: total length (femur, patella, tibia, metatarsus, tarsus). The terminology used in the text and figures follows Gabriel and Sherwood (2020).

A total of 366 bases of cytochrome oxidase I were sequenced by using the following primers: ExtA (5’-GAAGTTTATATTTTAATTTTACCTGG-3’) and ExtB (5’-CCTATTGAWARAACATARTGAAAATG-3’). This PCR profile consisted of an initial denaturing step at 94°C for 2 min, 30 amplification cycles [94°C for 30 s, 50°C or optimal annealing temperature (Tm°C) for 45 s, 72°C for 45 s], followed by a final extension step at 72°C for 5 min.

Materials from the following institutions were examined or had images of type material supplied to the authors: **IZCAS** Institute of Zoology, Chinese Academy of Sciences; **MHBU** Museum of Hebei University.

Abbreviations: **A** apical keel; **ALE** anterior lateral eyes; **AME** anterior median eyes; **BRV** base to receptacle value; **BSE** base separation extent; **CA** contraction area; **EO** embolic opening; **LBW** lobe to base width; **LRV** lobe to receptacle value; **LSE** lobe separation extent; **MOA** median ocular area; **NBW** neck to base width; **NLW** neck to lobe width; **PI** prolateral inferior keel; **PLE** posterior lateral eyes; **PLS** posterior lateral spinneret; **PME** posterior median eyes; **PMS** posterior median spinneret; **PS** prolateral superior keel; **SL** spermathecal lobes; **St** stalks; **TA** tegular apophysis.

### Comparative material examined

*Chilobrachysdominus* Lin & Li, 2022, holotype male, Ar42676, China, Ynnnan, Jinghong, IZCAS.

*Chilobrachysguangxiensis* (Yin & Tan, 2000), 2 males, without institution ID, China, Guangxi, Longzhou; 1 female, without institution ID, China, Hainan, Sanya, IZCAS.

*Chilobrachyshubei* Song & Zhao, 1988, 1 male, 1 female, without institution ID, China, Hubei, Badong (type locality), MHBU.

*Chilobrachysjinchengi* Lin & Li, 2022, holotype male, paratype male, Ar42677, Ar42678, respectively, China, Tibet, Medog, IZCAS.

*Chilobrachysliboensis* Zhu & Zhang, 2008, 1 male, without institution ID, China, Guizhou, Libo (type locality), IZCAS.

*Chilobrachyslubricus* Yu, Zhang, Zhang, Li & Yang, 2021, holotype male, one paratype female, without institution ID, China, Yunnan, Yuxi, MHBU.

## Taxon treatments

### Chilobrachys qishuoi

Lin & Li, 2022 sp. n.

7E59432F-1BD3-5103-90A0-9BE9D4C6B696

9B54BA15-963E-4244-9DB5-5D2426744C87

#### Materials

- **Type status:** Holotype. **Occurrence:** recordedBy: Shuo Qi and Jincheng Liu; sex: male; occurrenceID: E239F997-4C70-59CD-9731-D57BF718D5CC; **Location:** country: China; stateProvince: Guangdong; municipality: Qingyuan City; locality: Qingxin District, X366 Road, near Jingdong; verbatimElevation: 313 m; verbatimCoordinates: N24.2320°, E112.8048°; **Identification:** identifiedBy: Yejie Lin; **Event:** year: 2022; month: 9; day: 17–22; **Record Level:** institutionID: IZCAS-Ar43549
- **Type status:** Paratype. **Occurrence:** recordedBy: Shuo Qi and Jincheng Liu; sex: male; occurrenceID: B011EB7C-D71C-58FF-9649-488522763C14; **Location:** country: China; stateProvince: Guangdong; municipality: Qingyuan City; locality: Qingxin District, X366 Road, near Jingdong; verbatimElevation: 313 m; verbatimCoordinates: N24.2320°, E112.8048°; **Identification:** identifiedBy: Yejie Lin; **Event:** year: 2022; month: 9; day: 17–22; **Record Level:** institutionID: IZCAS-Ar43550
- **Type status:** Paratype. **Occurrence:** recordedBy: Shuo Qi and Jincheng Liu; sex: male; occurrenceID: B011EB7C-D71C-58FF-9649-488522763C14; **Location:** country: China; stateProvince: Guangdong; municipality: Qingyuan City; locality: Qingxin District, X366 Road, near Jingdong; verbatimElevation: 313 m; verbatimCoordinates: N24.2320°, E112.8048°; **Identification:** identifiedBy: Yejie Lin; **Event:** year: 2022; month: 9; day: 17–22; **Record Level:** institutionID: IZCAS-Ar43551
- **Type status:** Paratype. **Occurrence:** recordedBy: Shuo Qi; sex: female; occurrenceID: B011EB7C-D71C-58FF-9649-488522763C14; **Location:** country: China; stateProvince: Guangdong; municipality: Qingyuan City; locality: Qingxin District, X366 Road, near Jingdong; verbatimElevation: 313 m; verbatimCoordinates: N24.2320°, E112.8048°; **Identification:** identifiedBy: Yejie Lin; **Event:** year: 2022; month: 8; day: 20; **Record Level:** institutionID: IZCAS-Ar43552
- **Type status:** Paratype. **Occurrence:** recordedBy: Shuo Qi and Jincheng Liu; sex: female; occurrenceID: B011EB7C-D71C-58FF-9649-488522763C14; **Location:** country: China; stateProvince: Guangdong; municipality: Qingyuan City; locality: Qingxin District, X366 Road, near Jingdong; verbatimElevation: 313 m; verbatimCoordinates: N24.2320°, E112.8048°; **Identification:** identifiedBy: Yejie Lin; **Event:** year: 2022; month: 9; day: 17–22; **Record Level:** institutionID: IZCAS-Ar43553

#### Description

**Male** (holotype, IZCAS-Ar43549) (Fig. 2A, Fig. 3, Fig. 5, Fig. 6A, B and Fig. 7A).

Colouration in alcohol: Carapace, palp and legs red brown. Chelicerae and abdomen black (Fig. 3A).

Carapace 11.27 long, 9.82 wide, with long white setae. Eye group 2.02 long, 0.91 wide. MOA 1.24 long, anterior width 0.96, posterior width 1.24 (Fig. 3B). Eye sizes and interdistances: ALE 0.47, AME 0.42, PLE 0.38, PME 0.38; ALE–AME 0.11, AME–AME 0.18, PLE–PME 0.09, PME–PME 0.79. Fovea deep, slightly procurved, on one third of the carapace, occupying about one fifth of carapace width at that point. Four pairs of radial furrows (Fig. 3A).

Chelicera 6.94 long, 4.80 high, with long white grey setae. Promargin with dense brown hairs, retromargin with one row of 11 teeth, fang furrow with 35 denticles, strikers spiniform. Fang 5.54 long (Fig. 3G and H).

Labium 1.68 long, 2.21 wide, with 457 cuspules, covering almost 1/3 of area of labium, terminal brown, with long bristles (Fig. 3D).

Maxilla 4.73 long, 2.18 wide, with 267 cuspules. Stridulating lyra almost two times longer than height, with two kinds of stridulating setae: one row of 10 club-shaped, straight setae and dense spiniform setae (Fig. 3C, E and F).

Sternum 5.20 long, 4.47 wide, yellow brown, covered with two kinds of hairs: black erect bristles and non-erect brown hairs, separated from labium by fan-shaped areas. Three pairs of sigilla present, anterior pair small, oval; posterior pairs larger (Fig. 3D).

Legs with dense long and white setae on patellae and tibiae, without any spines. Tarsi I–III with 2 claws, tarsus IV with 3 claws, without denticle. Leg measurements: I 43.11 (12.46 + 4.92+ 11.17 + 8.57 + 5.99), II 37.47 (10.34 + 3.47 + 9.99 + 7.74 + 5.93), III 31.93 (8.07 + 3.28 + 8.09 + 8.12 + 4.37), IV 44.09 (11.57 + 3.45 + 11.55 + 11.92 + 5.60). Leg formula: 4123. Scopula on tarsus IV cracked by a band of macrosetae, scopulae of tarsi I–III not divided.

Abdomen 12.11 long, 6.37 wide, oval, without any pattern, covered with long light brown and white setae of varying lengths. PMS 1.75 long, PLS 8.99 long.

Palp (Fig. 5, Fig. 6A, B and Fig. 7A). Tibia with many setae laterally, swollen at base. Bulb oval, with tegular apophysis; embolus slightly curved, slender, sickle-shaped, with weakly developed apical, prolateral inferior and prolateral superior keels. Distal edge of embolus flat.

**Female** (one paratype, IZCAS-Ar43552) (Fig. 2B, Fig. 4 and Fig. 9d).

Colouration in alcohol: Same as in male (Fig. 4A).

Carapace 18.75 long, 16.54 wide, with long light brown setae. Eye group 6.41 long, 2.72 wide. MOA 2.19 long, anterior width 2.87, posterior width 4.45 (Fig. 4B). Eye sizes and interdistances: ALE 1.29, AME 0.98, PLE 1.24, PME 1.05; ALE–AME 0.66, AME–AME 0.71, PLE–PME 0.37, PME–PME 2.90. Others as in male (Fig. 4A).

Chelicera 10.68 long, 7.93 high, with long light brown setae. Promargin with dense brown hairs, retromargin with one row of 18 teeth, fang furrow with 94 denticles, strikers spiniform. Fang 8.68 long (Fig. 4G and H).

Labium 2.60 long, 2.98 wide, with 580 cuspules, others as in male (Fig. 4D).

Maxilla 8.02 long, 4.29 wide, with 580 cuspules. Stridulating lyra almost three times longer than its height, others as in male (Fig. 4C, E and F).

Sternum 8.48 long, 5.13 wide, others as in male (Fig. 4D).

Legs with long and short brown setae, others as in male. Leg measurements: I 52.92 (15.19 + 6.58+ 13.56 + 9.37 + 8.22), II 45.25 (13.09 + 5.90 + 11.21 + 8.67 + 6.38), III 40.12 (10.16 + 5.30 + 9.04 + 9.04 + 6.58), IV 51.89 (13.47 + 5.46 + 12.72 + 13.18 + 7.06). Leg formula: 1423.

Abdomen 20.36 long, 12.38 wide, covered with long light brown setae of varying lengths. PMS 2.75 long, PLS 11.87 long.

Spermathecae (Fig. 9d) simple. Two separated spermathecal lobes, without branch, middle with contraction, swollen distally, straight with genital furrow. Receptacles: 1.62 long, neck width 0.32, base width 0.71; contraction area: 0.46 long; spermathecal lobe: 0.50 long, 0.44 wide. LSE: 1.13 wide, BSE: 0.77 wide. BRV: 56%, LRV: 27%, LBW: 48%, NBW: 35%, NLW: 72%, LSE–BSE 68%.

#### Diagnosis

The new species is similar in habitus to *C.hubei*: the male with dense white setae on carapace, patellae and tibiae and the female with light brown carapace and chelicerae (Fig. 2A and B, Yu et al. 2021, figs. 3A and B). The bulbis similar to those of *C.hubei* and *C.liboensis* in having the same angle of the embolus relative to the bulb. The spermathecae are similar to those of *C.hubei* and *C.lubricus* by the spermathecal lobes swollen, with contraction at the neck, the base of the receptacles wider than the spermathecal lobes. The ratio of the length of the receptacle to BSE is almost 1:0.5 in *C.qishuoi* sp. n. and *C.hubei* and the length ratio of the spermathecal lobe to the contraction area is almost 1:1 in *C.qishuoi* sp. n. and *C.lubricus* (Fig. 9c, d, Yu et al. 2021, figs. 2C–E).

However, the male of *C.qishuoi* sp. n. can be distinguished from that of *C.hubei* by the apical keel with an angle range of 60° to 90° (Fig. 7) [vs. 120° in *C.hubei* (*Fig. 8c*) and 150° in *C.liboensis* (Fig. 8e)], the terminal of the embolus at a right angle in *C.qishuoi* sp. n. (Fig. 7) [vs. blunt in *C.hubei* and *C.liboensis* (Fig. 8c, e)] and the length ratio of the prolateral inferior keel to the apical keel almost 75%–80% in *C.qishuoi* sp. n. (Fig. 7) [vs. 62% in *C.hubei* (*Fig. 8c*) and 42% in *C.liboensis* (Fig. 8e)]. In females, the length ratio of the spermathecal lobes to the neck is almost 1:1 in *C.qishuoi* sp. n. (Fig. 9d) [vs. 1:0.25 in *C.hubei* (Fig. 9b, Yu et al. 2021, fig. 2F)], the receptacles are at 90° angle with genital furrow, with LBW almost 50% and the ratio of the length of the receptacle to the BSE almost 1:0.5 (Fig. 9d) [vs. 75° angle, LBW more than 50% and the ratio almost 1:0.3 in *C.lubricus* (Fig. 9c, Yu et al. 2021, figs. 2D and E)].

#### Etymology

The species is named after Mr. Shuo Qi, who collected type material; noun (name) in genitive case.

#### Distribution

Known only from the type locality (China, Guangdong) (Fig. 10).

#### Ecology

**Figure 1.**
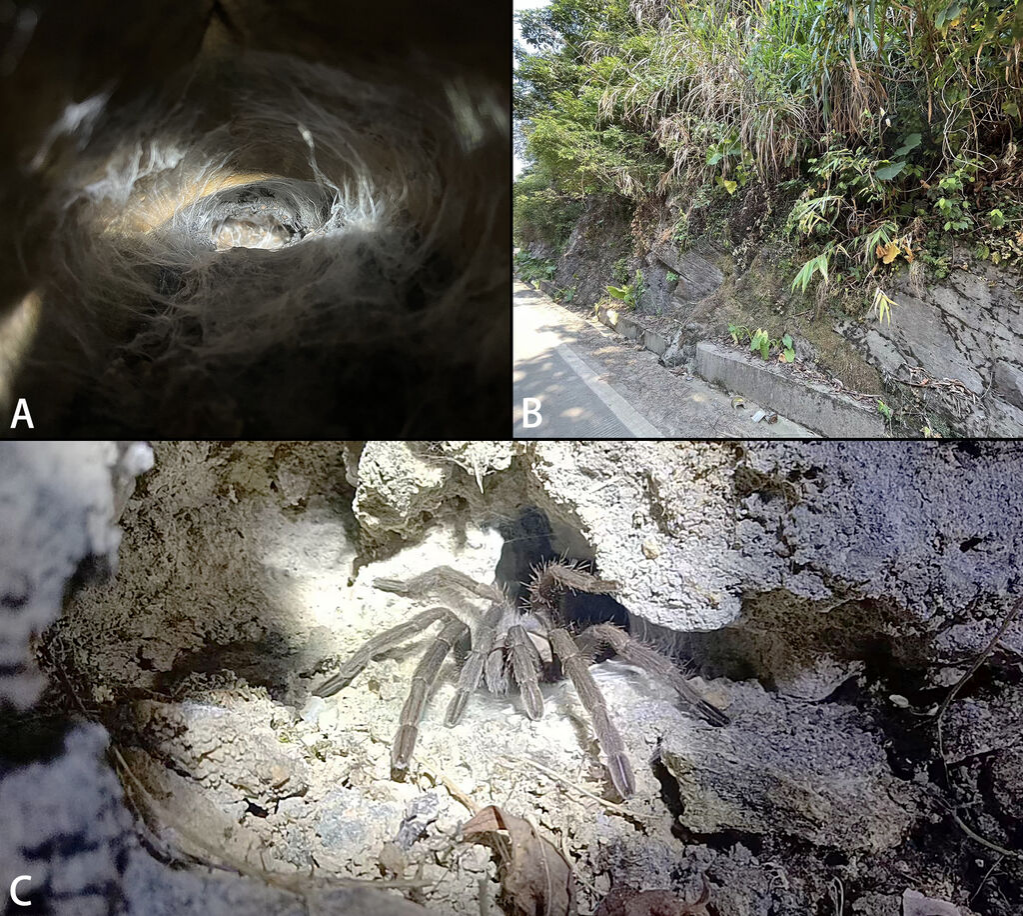
*Chilobrachysqishuoi* sp. n., live. **A** juveniles in burrow; **B** habitat in the type locality; **C** subadult, in situ. Photos by Shuo Qi (**A**, **B**) and Yongyou Zhao (**C**).

**Figure 2.**
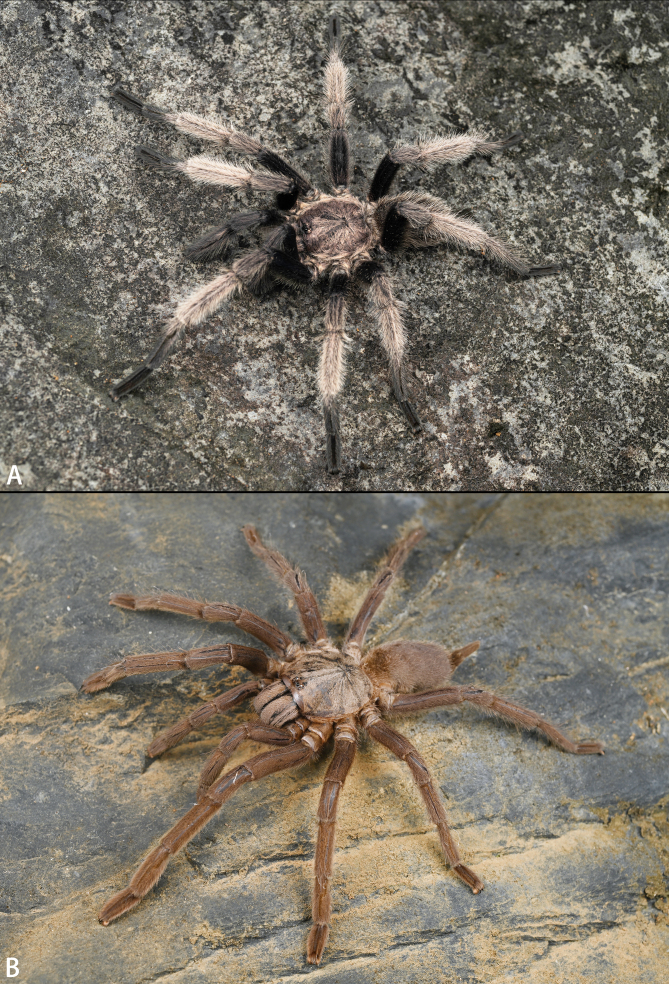
*Chilobrachysqishuoi* sp. n., **A** holotype male; **B** paratype female (Ar43552), live. Photos by Shuo Qi.

**Figure 3.**
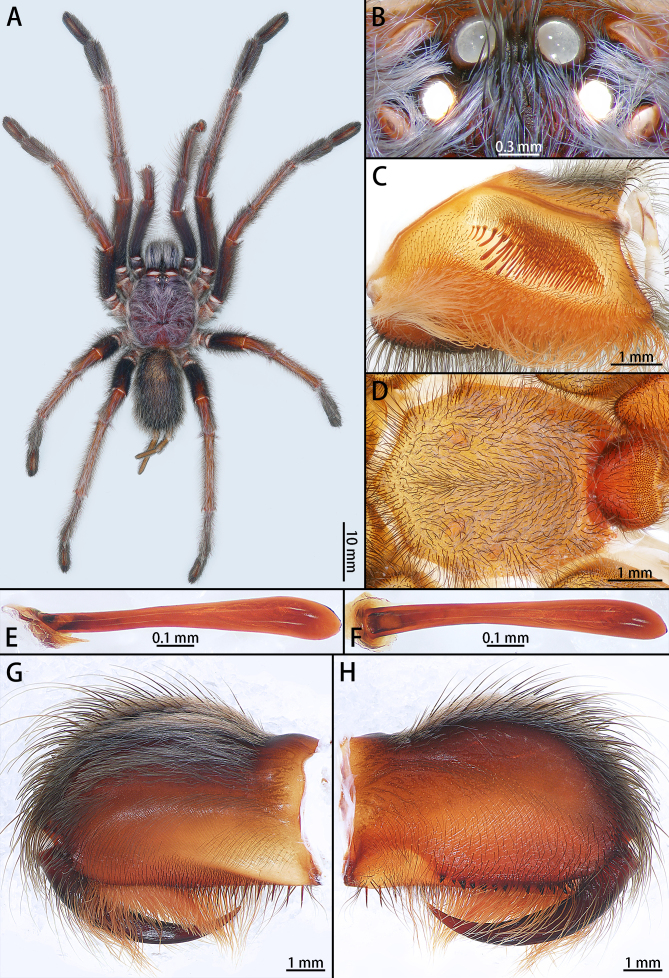
*Chilobrachysqishuoi* sp. n., holotype male **A** habitus, dorsal; **B** ocular tubercle; **C** left maxilla; **D** sternum; **E** stridulatory lyra, lateral view; **F** same, ventral view; **G** chelicera, retrolateral view; **H** same, prolateral view.

**Figure 4.**
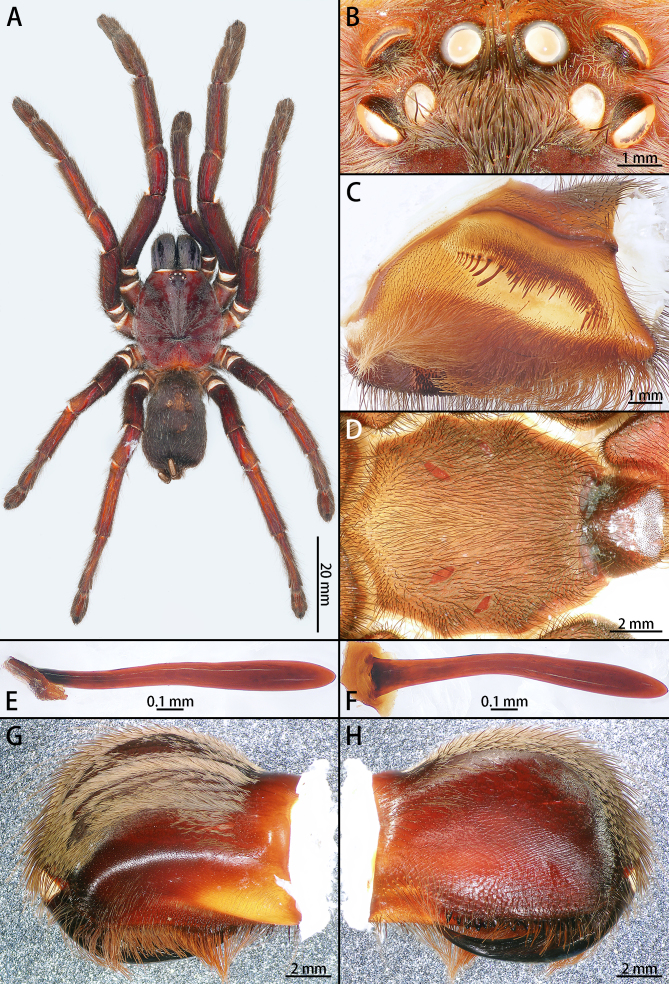
*Chilobrachysqishuoi* sp. n., paratype female (Ar43552) **A** habitus, dorsal; **B** ocular tubercle; **C** left maxilla; **D** sternum; **E** stridulatory lyra, lateral view; **F** same, ventral view; **G** chelicera, retrolateral view; **H** same, prolateral view.

**Figure 5.**
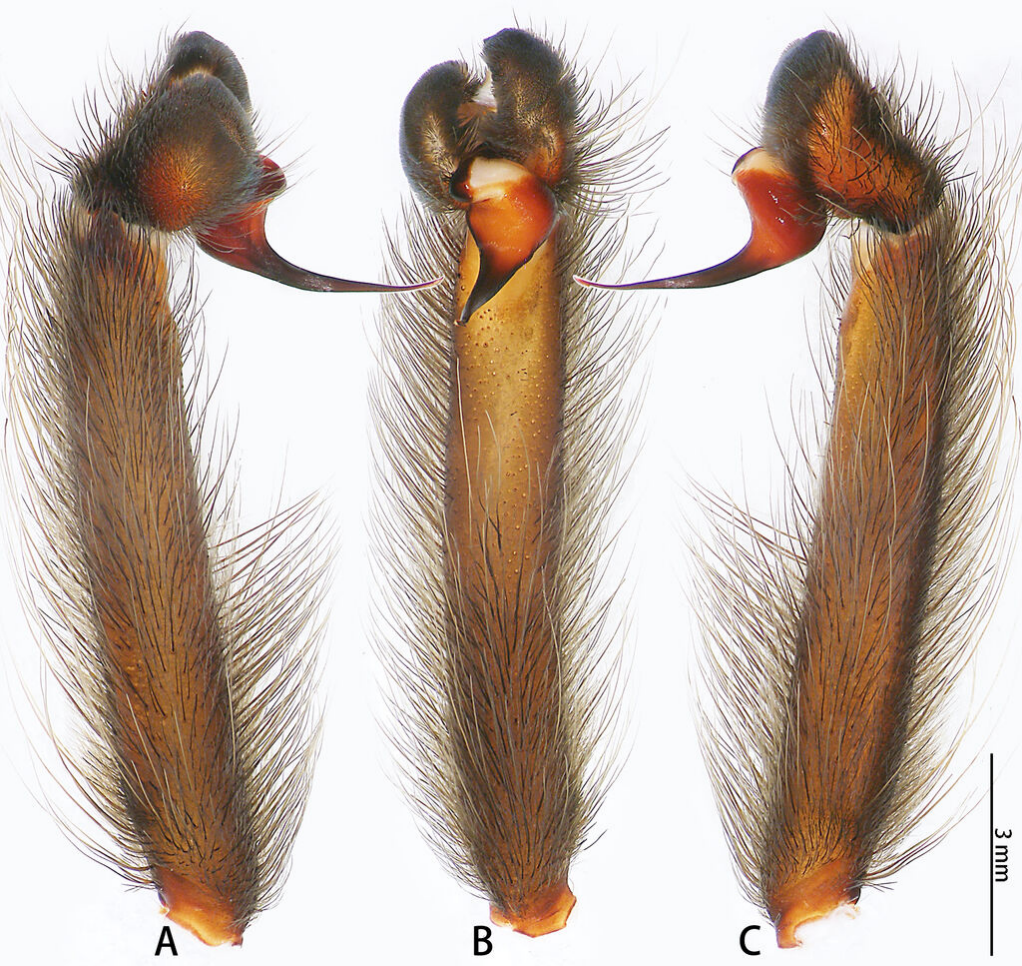
*Chilobrachysqishuoi* sp. n., holotype, male left palp. **A** prolateral view; **B** ventral view; **C** retrolateral view.

**Figure 6.**
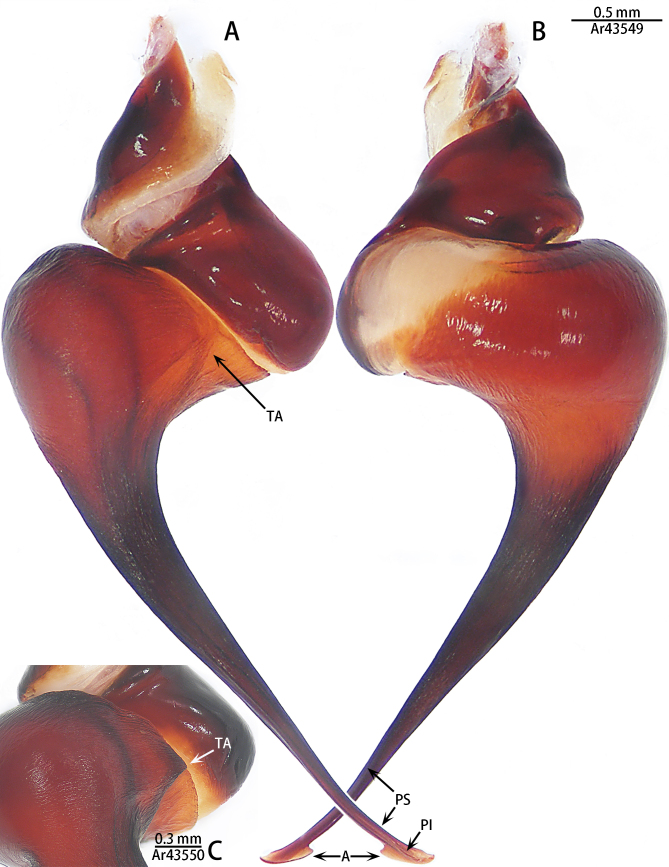
*Chilobrachysqishuoi* sp. n., right palp bulb, rotated horizontally **A**, **C** retrolateral view; **B** prolateral view. Abbreviations: **A** apical keel; **PI** prolateral inferior keel; **PS** prolateral superior keel; **TA** tegular apophysis.

**Figure 7.**
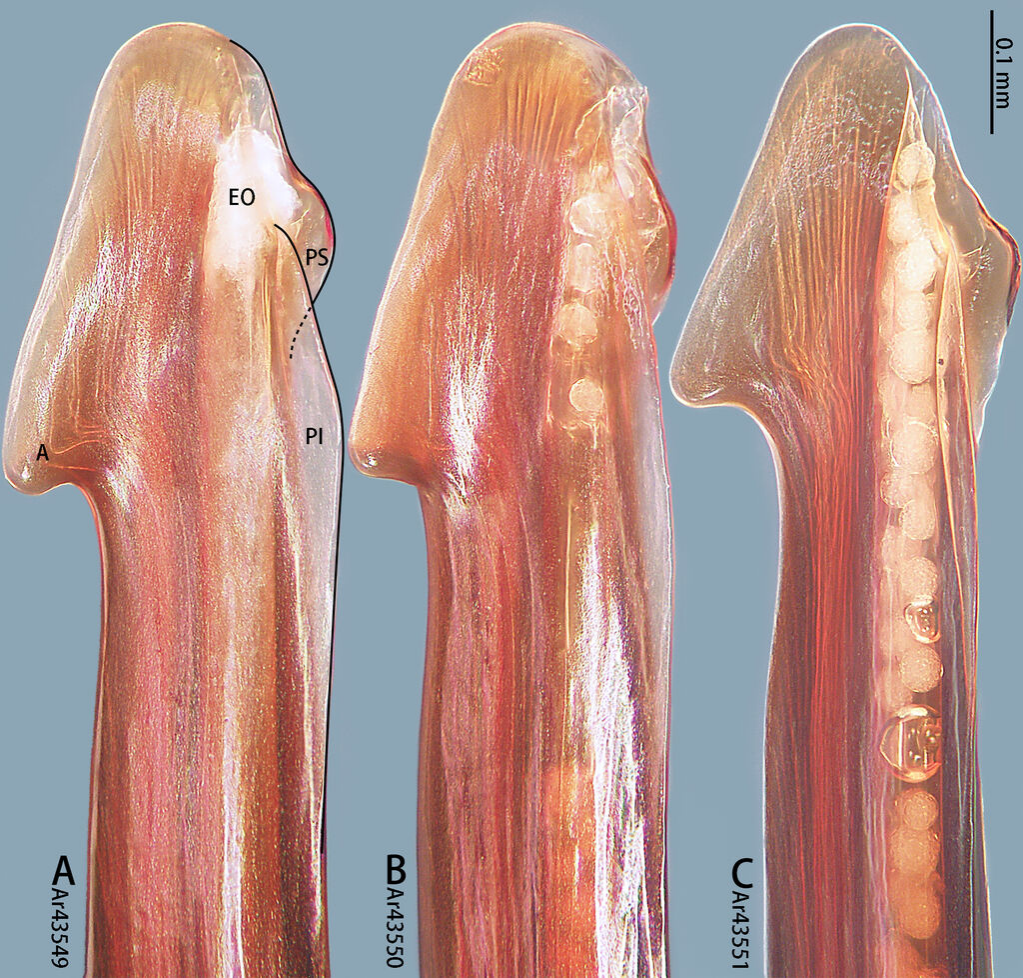
*Chilobrachysqishuoi* sp. n., tip of embolus. Abbreviations: **A** apical keel; **EO** embolic opening; **PI** prolateral inferior keel; **PS** prolateral superior keel.

**Figure 10.**
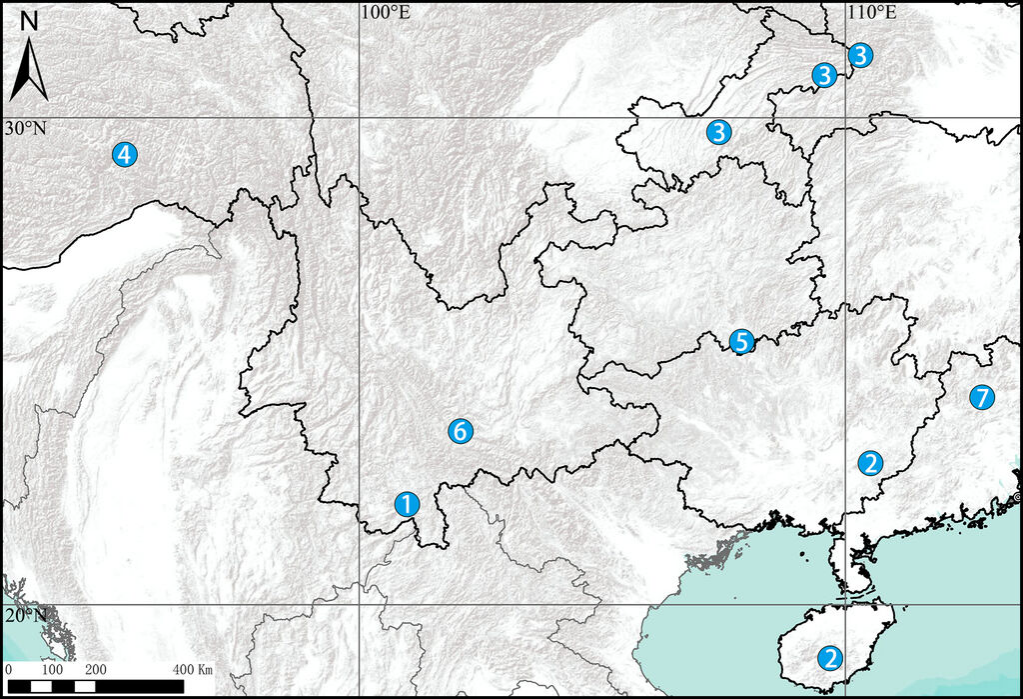
Distribution records of *Chilobrachys* species in China: **1** *C.dominus*; **2** *C.guangxiensis*; **3** *C.hubei*; **4** *C.jinchengi*; **5** *C.liboensis*; **6** *C.lubricus*; **7** *C.qishuoi* sp. n.

The specimens were found in barren limestone rock walls with some vegetation (Fig. 1B). They construct their retreats in natural rock burrows formed in the karst landscape; the burrows are usually about 6 to 8 cm in diameter. The web extends 20 to 30 cm inwards from the burrow (Fig. 1A). At night, they move to the entrance of the burrow, waiting for prey to pass by (Fig. 1C).

#### DNA barcode

CTATTATTAGATCATCTGTTGGGAAGCGTGAGCCCTTCGGAACTTTGGGAATAATTTATGCTATGGTTAGAATTGGTGGGATGGGGTTTGTTGTATGAGCTCATCATATGTTTTCTGTGGGAATAGATGTAGATACGCGGGCATATTTTACGGCAGCAACTATGGTGATTGCTGTCCCTACGGGAATTAGGGTATTTAGATGAATAGCTACGTTGTATGGATCTTACTTTAAGATGGATACCTCTTTGATATGGTGTGTTGGGTTCGTTTTTTTGTTTACTTTAGGGGGATTAACCGGGGTGGTTTTGGCTAATTCTTCTTTGGATATTATTTTGCATGATACTTATTATGTGGTTGCTCATTTTC (Ar43552, GenBank accession number OP394115).

## Supplementary Material

XML Treatment for Chilobrachys qishuoi

## Figures and Tables

**Figure 8a.**
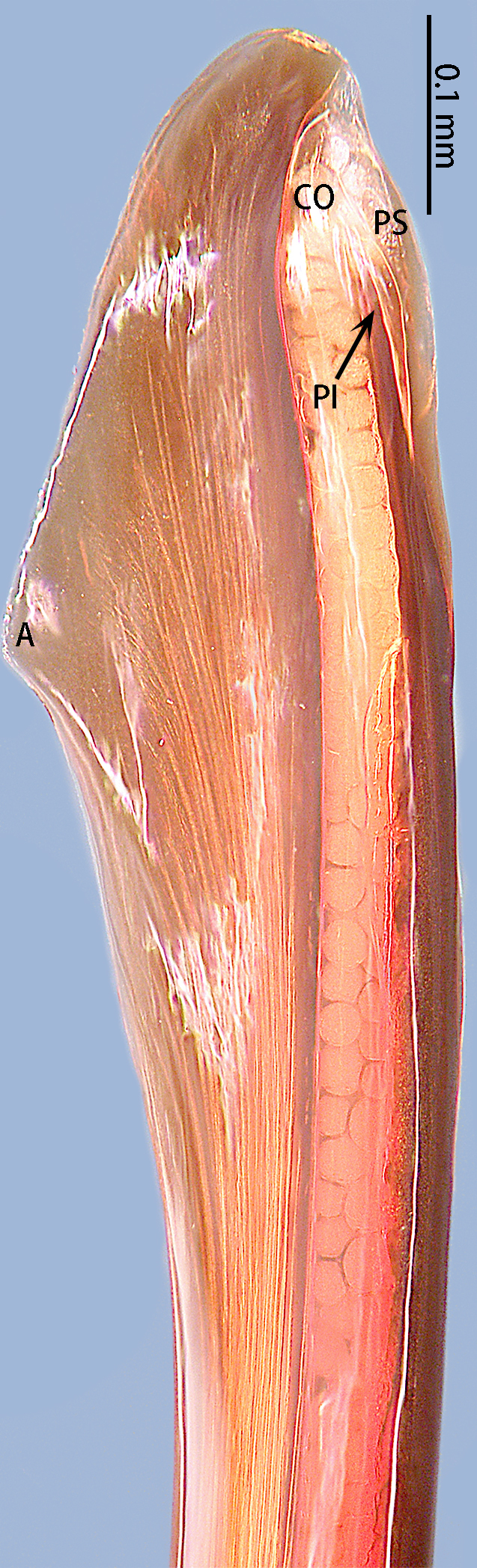
*C.dominus*, holotype

**Figure 8b.**
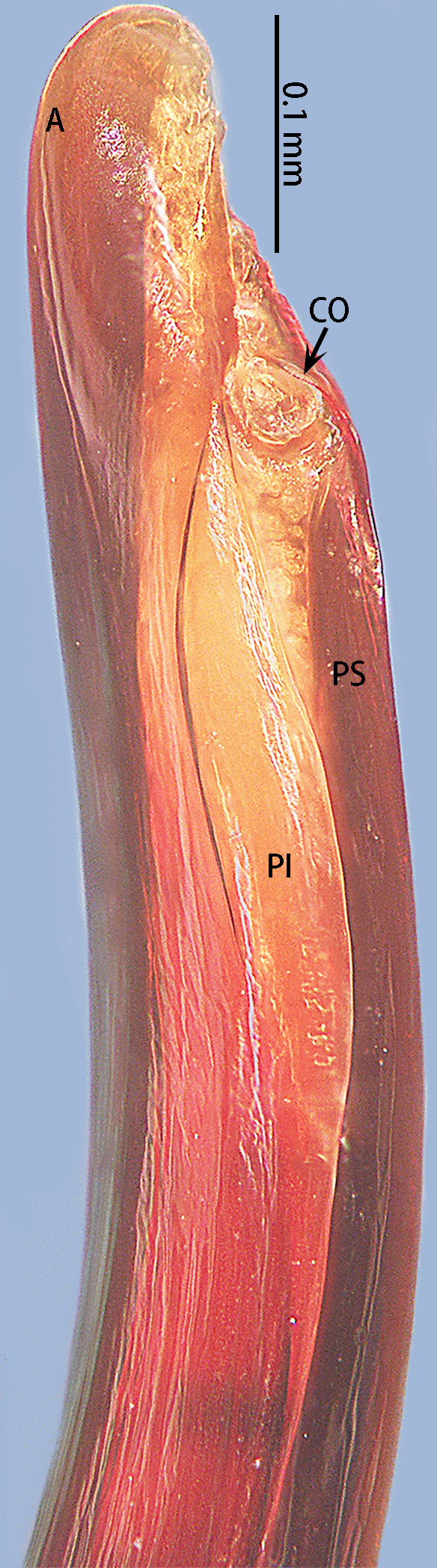
*C.guangxiensis* from Longzhou, Guangxi

**Figure 8c.**
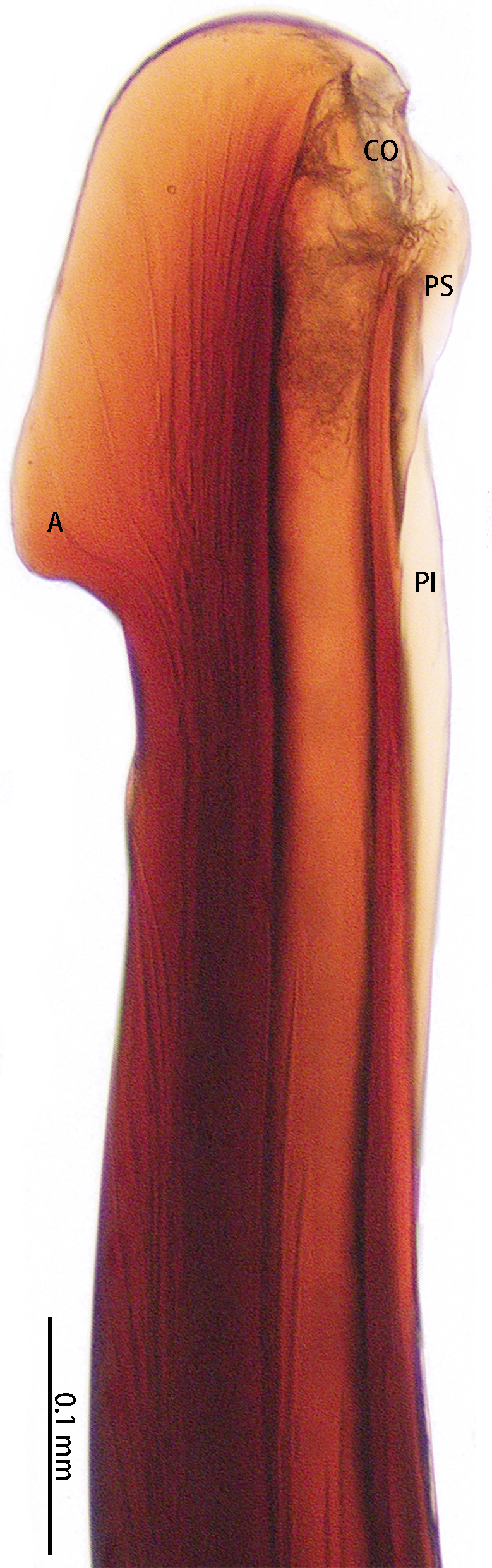
*C.hubei* from Badong, Hubei (type locality)

**Figure 8d.**
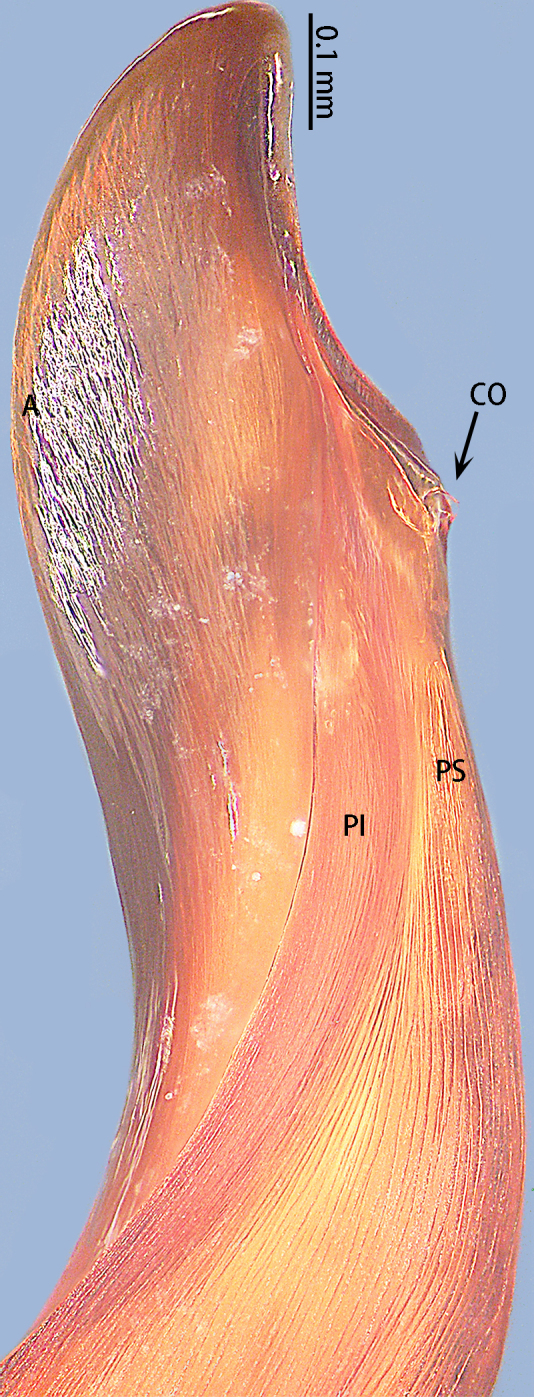
*C.jinchengi*, holotype

**Figure 8e.**
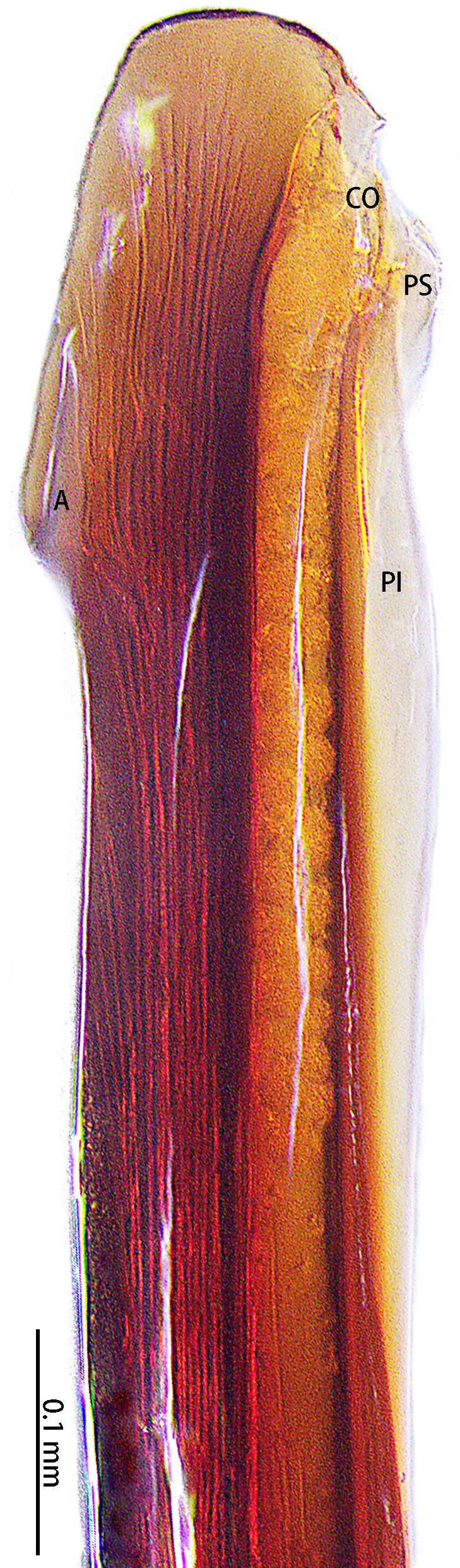
*C.liboensis* from Libo, Guizhou (type locality)

**Figure 8f.**
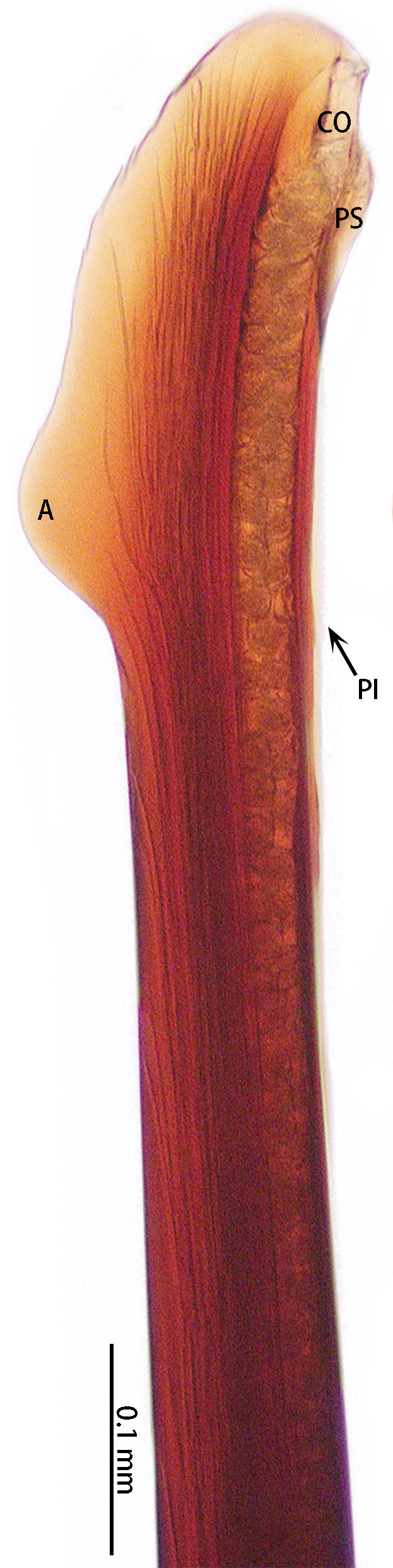
*C.lubricus*, holotype

**Figure 9a.**
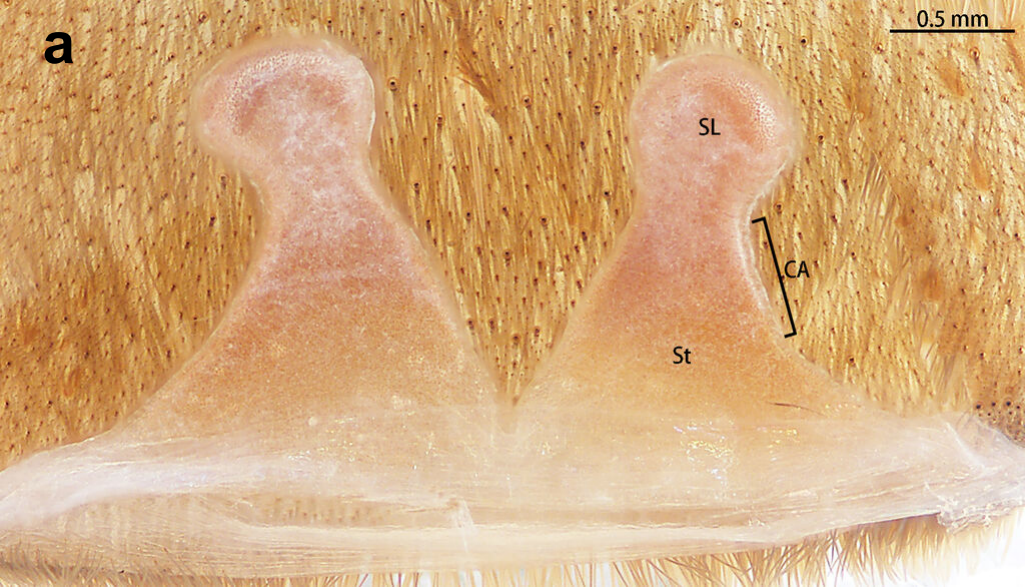
*C.guangxiensis* from Sanya, Hainan

**Figure 9b.**
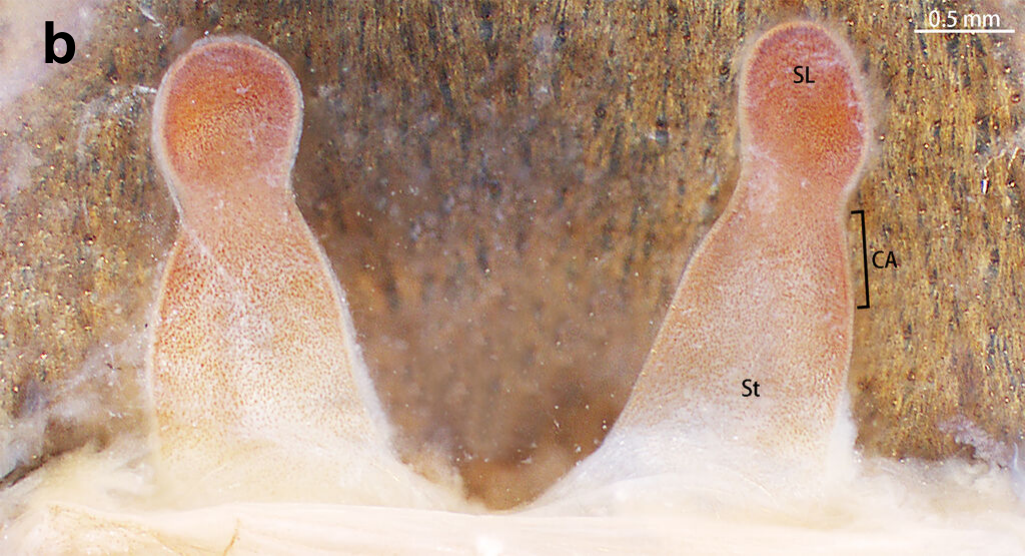
*C.hubei* from Badong, Hubei (type locality)

**Figure 9c.**
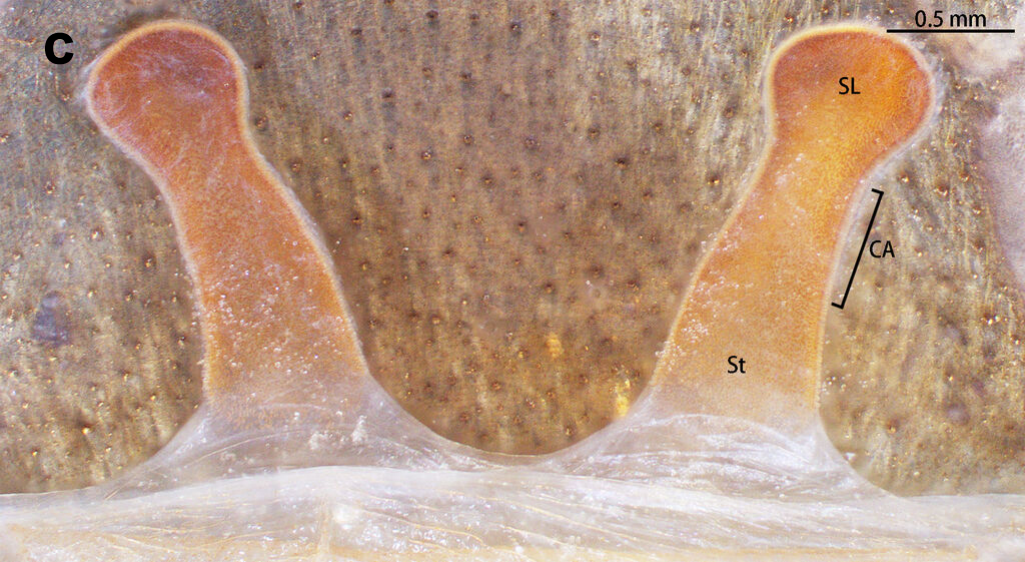
*C.lubricus*, paratype

**Figure 9d.**
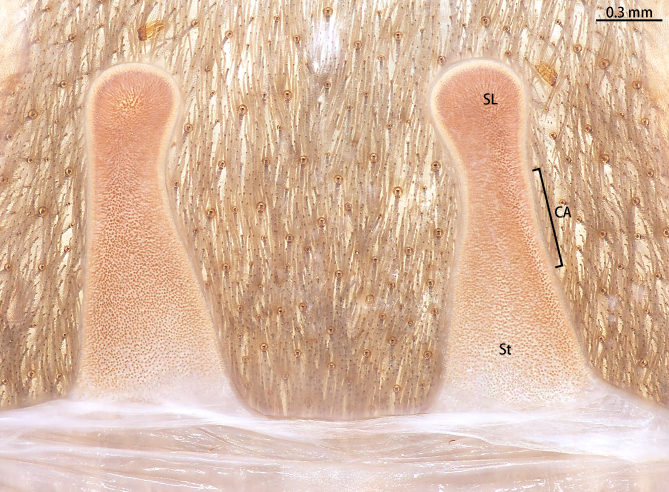
*C.qishuoi* sp. n., paratype (Ar43552)
